# Prebiotics and Synbiotics in Asthma: An Integrative Review of Human Trials and Murine Meta-Analysis

**DOI:** 10.3390/nu18040683

**Published:** 2026-02-20

**Authors:** Louise C. Bonnard, Graham R. Sharpe, Matthew Martin, Georgina F. Dodd, Neil C. Williams

**Affiliations:** 1 Department of Sport Science, Nottingham Trent University, Nottingham NG11 8NS, UK; graham.sharpe@ntu.ac.uk (G.R.S.); neil.williams@ntu.ac.uk (N.C.W.); 2 Respiratory Clinical Research Facility, Nottingham Respiratory Research Unit, City Hospital, Nottingham University Hospitals NHS Trust, Nottingham NG5 1PB, UK; matthew.martin5@nottingham.ac.uk; 3 Clasado Biosciences Ltd., Imperium Building, Imperial Way, Worton Grange, Reading RG2 0TD, UK; georgina.dodd@clasado.com

**Keywords:** asthma, prebiotics, synbiotics, airway inflammation, systemic inflammation, microbiota

## Abstract

**Background/Objectives:** The objectives of this study were to systematically review the literature on the effects of prebiotics and synbiotics on asthma control, lung function and asthma-associated inflammation from murine and human trials. **Methods:** A systematic review was performed following the PRISMA guidelines across multiple databases. A meta-analysis was performed on murine trials assessing asthma-associated inflammation and airway hyperresponsiveness, whilst a narrative review of human studies assessed asthma control, lung function, and inflammation. **Results**: Seventeen studies met the eligibility criteria for inclusion; eleven murine studies were included for meta-analysis and six human studies were for narrative review. The meta-analysis revealed significant effects of prebiotics and synbiotics on multiple markers of asthma-associated inflammation. Prebiotic intervention significantly reduced airway hyperresponsiveness (AHR) and type 2 cytokines (IL-4, IL-5, IL-13) and various cell counts, including neutrophil, macrophage, lymphocyte, eosinophil, and total bronchoalveolar (BALF). Synbiotics were also effective in reducing type 2 cytokines, including, IL-4, IL-5, IL-13, and lymphocytes, eosinophils, and total BALF cell count. A narrative review of human intervention trials of prebiotics and/or synbiotics revealed improvements in lung function, asthma control, and systemic and airway inflammation. **Conclusions:** This review indicates that dietary prebiotics and synbiotics may be suitable adjunct treatments to support asthma management, but further well-controlled human RCTs are required.

## 1. Introduction

Asthma is a common inflammatory respiratory disease of the airways, affecting more than 300 million people globally and causing around 1000 preventable deaths daily [1]. The economic burden of asthma continues to increase for healthcare systems, communities and individuals [2]. In the UK alone, the estimated total direct cost of asthma is £1.5 billion [3], and in Europe, the asthma-related economic burden is estimated at €19 billion [4]. Although there are multiple effective treatments for asthma, including inhaled corticosteroids and short- and long-acting β_2_-agonists, they have a limited effect on the underlying pathology and inflammation associated with asthma and do not modify disease progression [5]. Approximately 50% of mild-to-moderate asthma, and a considerable proportion of severe phenotypes, are driven by Type 2 (Th2)-high inflammation [6]. In this process, Th2 lymphocytes recognise allergen presentation and secrete Th2 pro-inflammatory cytokines including IL-4, IL-5 and IL-13. This cascade leads to eosinophil recruitment and IgE production, and, subsequently, the airway smooth muscle becomes sensitised, resulting in increased mucus secretion and bronchial hyperresponsiveness [7]. Due to the high morbidity and socioeconomic burden of asthma, interest in adjunct treatments to support disease management has increased [8]. With evidence that the gut microbiome may differ in asthma patients [9] and of the role of the gut microbiome in modulating inflammation and immunity [10], strategies that target the microbiome to support asthma management are warranted.

The benefits of probiotics for asthma have been systematically reviewed [11,12]. Prebiotics and synbiotics have received growing interest for their effects on asthma, recognised for their minimal adverse effect profile, low cost, and wide availability [12]. A probiotic is a live microorganism that, when administered in adequate amounts, confers a health benefit on the host, and a prebiotic is a substrate that is selectively utilised by host microorganisms conferring a health benefit [13]. Synbiotics are a combination of live microorganisms (probiotics) and substrate(s) (prebiotics). Synbiotics can be administered as a simple probiotic and prebiotic combination—a ‘complementary synbiotic’—or as a ‘synergistic synbiotic’—prebiotics selectively utilised by the co-administered microorganisms to confer additive benefits.

Previous murine studies report anti-inflammatory and protective effects on the airway hyperresponsiveness of various prebiotics, including raffinose, galacto-oligosaccharides (GOS), fructo-oligosaccharides (FOS), and pectin-derived acidic oligosaccharides (AOS) [14,15,16]. Clinical interventions often supplement galacto-oligosaccharides (GOS) and fructo-oligosaccharides (FOS), typically in a 9:1 ratio formulated to mimic the natural distribution and prebiotic effects of human milk oligosaccharides (HMOs) [17]. HMOs are complex glycans synthesised from lactose in the mammary gland and they function as bioactive compounds that are critical for early-life microbiota establishment and the maturation of the immune system [18]. HMOs have been recognised to protect against allergic disease [19] via direct immunomodulatory effects on the intestinal mucosa and systemic immune cells [20]. These oligosaccharides can additionally be found in natural sources such as fruits, vegetables, and honey, which also contribute to the colonisation of the gut microbiome and improvement of the immune response [21]. Although emerging evidence supports a wide variety of non-carbohydrate prebiotics, including (poly)phenols and polyunsaturated fatty acids [22], this review highlights the benefits of carbohydrate-based prebiotics. These represent the most extensively established evidence base in the modulation of respiratory health and asthma pathophysiology.

Promising findings in murine asthma research have generated interest in the use of prebiotics and synbiotics in humans. Although human studies are limited, data highlights improvements in lung function and reductions in markers of systemic inflammation [23], airway hyperresponsiveness [24], and the alleviation of asthma symptoms [25] following prebiotic and synbiotic treatment. Emerging evidence implicates the gut microbiome as a key modulator of the asthma-associated inflammatory cascade, where a lack of microbial diversity or ‘dysbiosis’ is closely linked to respiratory disease [26]. Specifically, beneficial gut bacteria are often less abundant in asthma patients [27], while pathogenic strains are often enriched [28].

Whilst the mechanisms by which the gut–lung axis may mediate respiratory disease remain unclear, it has been suggested that an enriched gut microbiota can mediate these processes via interaction with intestinal epithelial and dendritic cells. These cells sample luminal bacteria and induce molecular responses that polarise T cells towards T-regulatory cells, thereby moderating the asthma-associated inflammatory response [29]. Furthermore, the metabolism of dietary prebiotics by commensal bacteria produces short-chain fatty acids (SCFAs), such as butyrate, which can enter systemic circulation and exert inhibitory effects on lung inflammation [30].

A recent review describes the use of prebiotics and synbiotics in a variety of immune-mediated conditions [31]. Another review describes prebiotic and synbiotic use in paediatric asthma [32]. However, the effects of prebiotics and synbiotics for the primary treatment of asthma symptoms, asthma control and inflammatory outcomes in adults and children have not been reviewed. We therefore sought to review the relevant literature on murine and human randomised controlled trials (RCTs) to assess our hypothesis that prebiotics and synbiotics may elicit immunomodulatory effects, including the reduction in type 2 markers and allergic airway inflammation. Additionally, through a narrative analysis of existing human studies, we sought to identify the study elements that can inform the design of future human RCTs and address unanswered questions in this field.

## 2. Materials and Methods

### 2.1. Reporting Guidelines and Protocol Registration

The protocol for this systematic review was registered with PROSPERO (National Institute for Health Research, University of York, UK), accessed online at https://www.crd.york.ac.uk/prospero/display_record.php?ID=CRD42024536669 (accessed on 1 January 2026). Register number: CRD42024536669. This review was performed according to a prospective protocol for systematic reviews and meta-analysis (PRISMA).

### 2.2. Search Strategy

The electronic databases Web of Science, Pubmed, Cochrane Library, and Scopus were searched for their eligible studies from 1970 to 1 February 2026. The search strategy was based on the guidance of the ‘Cochrane handbook’. The search formulas of the databases were according to the following forms: (Prebiotic OR synbiotic OR oligosaccharide* OR galactooligosaccharide* OR GOS OR fructooligosaccharide* OR FOS OR “soluble fibre” OR inulin OR raffinose OR synbiotic*) AND (asthma OR “airway inflammation” OR “hyper responsiveness” OR “allergic airway inflammation” OR “bronchial asthma” OR “allergic airway eosinophilia”) AND (murine OR mice OR rat* OR adult* OR child* OR infant*).

#### 2.2.1. Inclusion and Exclusion Criteria

Inclusion and exclusion criteria for this review were developed considering Population, Intervention, Comparison and Outcome (PICO) [33].

#### 2.2.2. Population

Human participants in this review include children and adults diagnosed with asthma. The inclusion of human participants was not limited by asthma phenotype/aetiology, age, gender or ethnic group.

Murine studies include mice and rat asthma-induced models as part of a randomised-control trial (RCT) of any duration.

#### 2.2.3. Intervention

For inclusion, human studies had to include the administration of a prebiotic or synbiotic treatment of any duration. No restrictions were placed on the dose of prebiotic and synbiotic intervention.

For the included murine studies, no restrictions were placed on the type of allergen, type of sensitisation, duration of sensitisation, or allergen challenge employed in allergic-asthma murine models.

#### 2.2.4. Comparison

Murine Studies were required to have a comparative control. Human studies were RCTs and used an appropriate placebo or control, and were either cross-over in design (participants acted as their own control) or part of a parallel-design (subjects received either treatment or placebo).

#### 2.2.5. Outcome

Murine studies were only included if they used allergic asthma models and if the outcome measures included airway hyperresponsiveness, cytokines or cells, or total bronchoalveolar lavage fluid (BALF) cell counts, including its white blood cell constituents (eosinophils, neutrophils, lymphocytes and alveolar macrophages). Human studies were only included if at least one assessment of lung function, markers of inflammation, or subjective markers of asthma control and symptoms were assessed.

Data for the meta-analysis were included if mean and standard deviations were reported, allowing for the calculation of the standardised mean difference (SMD) between the prebiotic/synbiotic intervention and comparator. Where possible, differing units of measurements were converted into the same units of comparison (gold standard).

### 2.3. Study Selection

**Figure 1:**
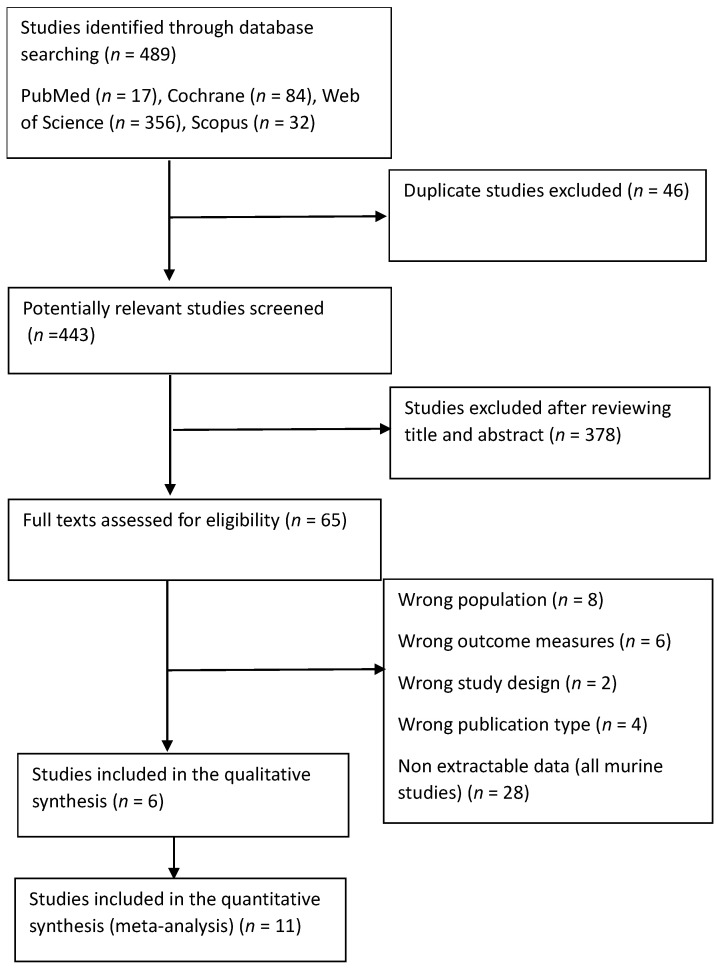
Preferred reporting items for systematic reviews and meta-analyses flowchart of articles included in the systematic review of the effect of prebiotics and synbiotics on asthma outcome measures.

A two-stage process was completed for the screening and selection of studies. Articles retrieved through the systematic search were exported to the reference management software ProQuest RefWorks (RefWorks 3.0, Pro-Quest LLC, Ann Arbor, MI, USA) and to Excel (Microsoft 365, Microsoft, Washington, DC, USA) in order to remove duplicates and assess for eligibility. Articles were independently screened by two investigators (L.B. and N.W.) in accordance with eligibility criteria based on the title and abstract. Full texts from eligible studies were then independently screened (L.B. and N.W.) for inclusion in the review (Figure 1).

### 2.4. Data Extraction and Management

Study characteristics were extracted by one reviewer (LB) using a self-designed Excel data extraction sheet. Extracted data included the first author’s name, title, publication details, participant characteristics (age, sex, asthma diagnosis/induction, pre-study control measures including medication usage), sample size (experimental and control group), geographic area of conducted research, characteristics of biomarker information (biomarker assessment and unit of measurement) and details of the prebiotic/synbiotic intervention were extracted. Where appropriate, data was not presented, and the authors were emailed and allocated 4 weeks to reply. If no reply was received, an alternative approach of manually measuring and extracting data from graphical figures was utilised. If this was not possible or was deemed unclear, the study was excluded from meta-analysis. Any variables included in the search string that did not have sufficient commonality with other studies (human interventions) were presented in the narrative review.

### 2.5. Assessment of Methodological Quality

The quality assessment of murine studies was conducted using the SRCYLE’s (Systematic Review Centre for Laboratory animal Experimentation), a Risk of Bias tool designed to establish consistency and avoid discrepancies in assessing the methodological quality of Animal RCTs and experimentation [34].

The risk of bias for human studies was completed using the Cochrane Handbook for Systematic Reviews of Interventions, in accordance with ‘Chapter 8: Assessing risk of bias in a randomised trial’ in the *Cochrane Handbook for Systematic Reviews of Interventions* [35] [Online]. Study components were assessed using the ROB-2 tool which included (i) the randomisation process, (ii) deviations from intended interventions, (iii) missing data, (iv) measurement of outcomes, and (v) reporting of results. All items were graded as ‘low’, ‘high’, or ‘unclear’ risk of bias.

### 2.6. Statistical Analysis

A narrative synthesis was undertaken where quantitative pooling was considered inappropriate due to insufficient amount of data for the meta-analysis of human studies.

For murine data, an inverse variance random effects meta-analysis was conducted on airway hyperresponsiveness and markers of inflammation using the Review Manager Software (RevMan, Version 5.3, Cochrane Collaboration, Oxford, UK). Hedge’s g standardised mean difference (SMD) was calculated via the RevMan software (RevMan, Version 5.3, Cochrane Collaboration, Oxford, UK).

Separate meta-analyses were conducted for airway hyperresponsiveness and biomarkers of inflammation, which used the same units and method of assessment. As cytokines varied in source, data was normalised relative to placebo/control prior to meta-analysis, and data were presented as standardised mean difference (SMD) for all parameters. Effect size was calculated using Hedges’ g, employing an inverse variance approach with 95% confidence interval (CI). Classification included very small (0.01–0.19), small (0.20–0.49), moderate (0.50–0.79), large (0.80–1.19), very large (1.20–1.99) and huge (>2.0) [30]. The I-squared test (*I*^2^) was carried out to determine the extent of statistical heterogeneity; 50–75% indicated substantial heterogeneity and 75–100% indicated considerable heterogeneity (Cochrane). All results were reported as Hedges’ g with 95% confidence intervals (CIs).

## 3. Results

A total of 489 studies were included in the electronic searching, and 443 remained after duplicates were removed (Figure 1). Following this, 378 of the 443 were deemed ineligible following review of the title and abstracts. The full texts of the remaining 65 were assessed by two reviewers, eight of which were excluded based on population, six excluded based on reporting of outcome measures, and two excluded based on study design. Four studies followed a RCT design but were conference proceedings and not eligible for this review, and 28 provided non-extractable data (authors were contacted, but no response was received). No studies were found via manual search. A total of 17 studies met the final inclusion criteria: 11 murine studies eligible for meta-analysis and six human studies eligible for a narrative review.

### 3.1. Risk of Bias

#### 3.1.1. Risk of Bias—Murine Studies

**Figure 2:**
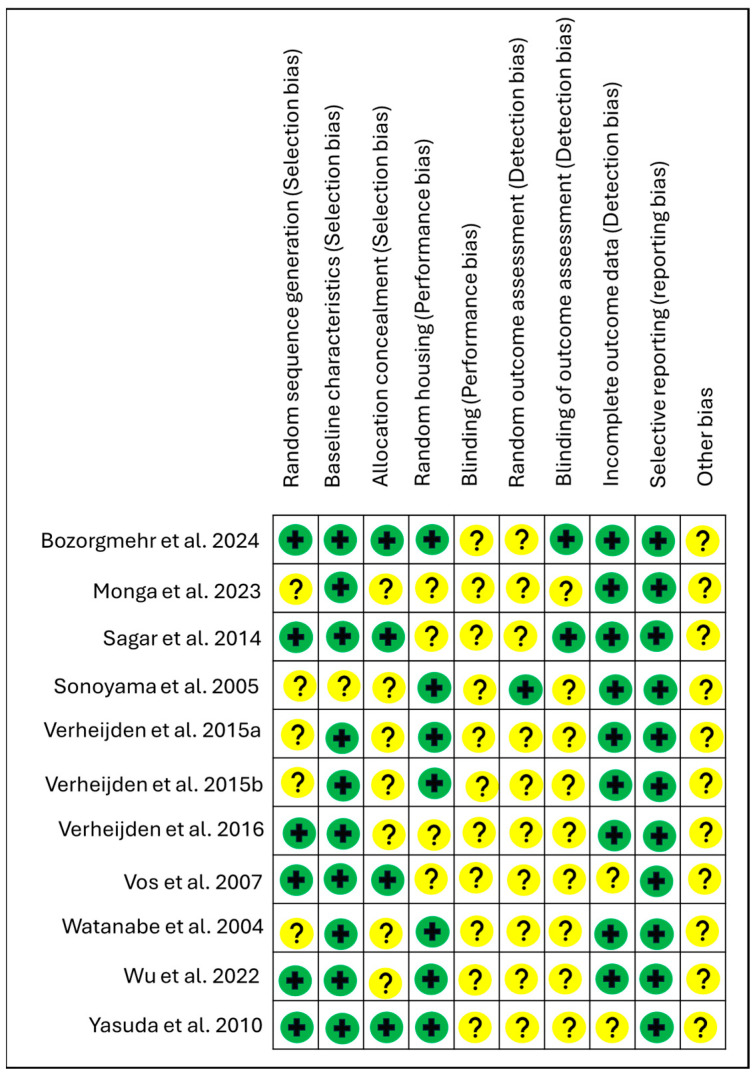
SYRCLES’s Risk of bias summary: review author’s judgements about each risk of bias item for each included study [14,15,16,36,37,38,39,40,41,42,43]. Green indicates low risk of bias (±); Yellow indicates unclear risk of bias (?).

**Figure 3:**
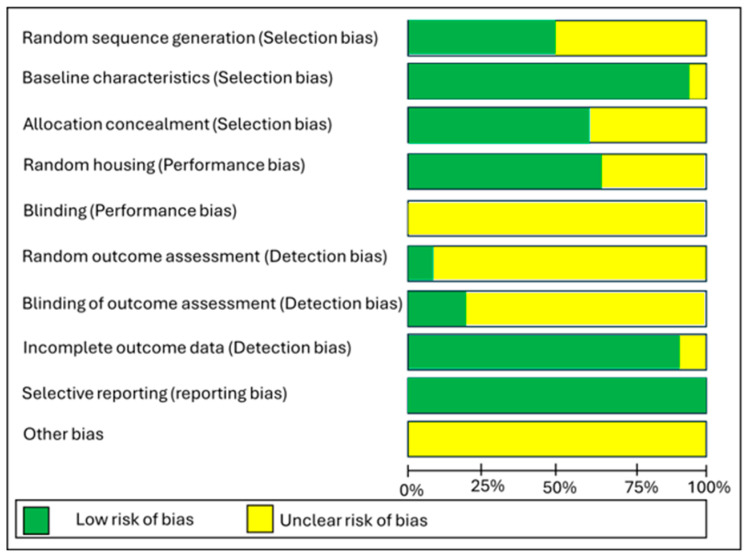
SYRCLE’s Risk of bias graph: review author’s judgements about each risk of bias item presented as percentages across all included studies.

The risk of bias for murine studies was assessed using the SYRCLE tool, designed for the assessment of risk of bias in animal studies [34]. Risk of bias in different domains for each included study is summarised in Figure 2, and each risk of bias item is presented as percentages across all included studies in Figure 3. In the majority of studies, allocation concealment, blinding of the caregiver/investigator/outcome assessor, random housing conditions and random outcome assessment were poorly described. Most studies were at low risk for baseline characteristics, random housing, incomplete outcome data and selective reporting.

#### 3.1.2. Risk of Bias—Human Studies

A complete analysis of risk of bias in the human studies is displayed in Table 1. Risk of bias was assessed by one reviewer (LB) and verified by a member of the review team (NW). The Cochrane collaboration tool ROB-2 was used to cover all human randomised controlled trials (Cochrane Collaboration 2021; Oxford, UK). The ROB-2 assessed components of the studies, including the randomisation process, deviations from intended interventions, missing outcome data, measurement of outcomes, and reporting of results. Bias due to measurement of the outcome was judged as low for all human studies (*n* = 6), and risk of bias due to missing outcome data was judged as low for most human studies (*n* = 5).

A ‘some concerns’ rating due to the reported randomisation process and deviations from intended deviations was given for Halnes et al. [44], as there was limited detail on participant randomisation to the prebiotic and placebo intervention; furthermore, there were limitations with intervention design where treatment was not taste or appearance matched (synbiotic yoghurt compared with mashed potato placebo). Bias due to the risk of bias in the measurement of the outcome was rated as ‘low’ for 6 studies, considering that standard scientific procedures were often described and most outcome measures were objective; therefore, it was unlikely that assessor interpretation would affect outcome data (Table 1).

**Table 1:** Risk of Bias assessment of included studies using Cochranes ROB-2: Low (✓), Some concern (~).

ROB-2						
Study	Domain 1	Domain 2	Domain 3	Domain 4	Domain 5	Overall ROB
	Risk of Bias arising from randomisation process	Risk of Bias due to deviations from intended interventions	Risk of Bias due to missing outcome data	Risk of Bias in measurement of the outcome	Risk of Bias in selection of reported result	
Human						
Berthon et al. (2025) [45]	✓	✓	✓	✓	✓	✓
Halnes et al. (2017) [44]	~	~	~	✓	~	~
Hassanzad et al. (2019) [25]	✓	~	✓	✓	✓	~
Mcloughlin et al. (2019) [30]	✓	✓	✓	✓	✓	✓
Van De Pol et al. (2011) [23]	~	~	✓	✓	~	~
Williams et al. (2016) [24]	✓	✓	✓	✓	✓	✓

### 3.2. Findings from Animal Studies

#### 3.2.1. Description of Included Murine Studies

Eleven murine studies were included in the meta-analysis (Table 2). Seven studies investigated the effects of prebiotic treatment and four studies investigated synbiotic treatment. The publication year ranged from 2004 to 2024.

Studies used various strains of laboratory murine models, including C57BL/6J SPF mice [36], BALB/c mice [15,16,18,37,38,39], brown Norwegian rats [14,40] and C3H/HeN mice [41]. Six studies used ovalbumin-exposure [16,37,39,40,42] and five [15,36,41,43] used house dust mites to induce allergic airway inflammation (AAI). Two studies performed a sensitisation in combination with the Bordetella Pertussis vaccine [14,40]. The initial allergen exposure ranged from day 0 to day 63 (median = 31.5). In three studies, an airway hyperresponsiveness (AHR) test was conducted [38,42,43] between day 14 and 70 (median 20), with AHR expressed as the enhanced pause parameter [42] or average airflow resistance (cm H_2_O/mL/s) [38,43].

#### 3.2.2. Control Interventions

The control treatments for studies were either a specific control diet [36,39,41] or a control treatment (AIN-93G) formulated by the American institute of Nutrition [14,38,40,42,43] or OVA control treatment [37], or was unspecified [16].

#### 3.2.3. Experimental Interventions

Across the eleven murine studies, the intervention treatment duration ranged from 7 to 55 days (median = 31) ( Table 2). GOS was the most common type of prebiotic, used (in combination/alone) by 6 (54%) studies [14,15,38,39,42,43]. Five (45%) used FOS [16,39,40,41,42], two (18%) used RAF [14,40], one (9%) used XOS [40], 1 (9%) used pOAS (pectin-derived oligosaccharides) [42], one (9%) used Sucosyllactose and fucosyllactos [36], and one (9%) used Isomaltooligosaccharides [37]. Of the four synbiotic studies, two studies combined the probiotic *B. breve* M16V with the aforementioned prebiotics [16,38], one study treated with *Bifidobacterium breve Bif11* MTCC25246 and *Lactiplantibacillus Plantarum LAB31* [37], and the final study used *Lactiplantibacillus acidophilus* and *B.animalis* [39].

#### 3.2.4. Multi-Trial Studies

Four murine studies carried out several trials with various doses/types of prebiotics/synbiotics. Monga et al. [37] used a low dose (Tx 1) (1 × 10^10^ CFU) and a high dose (Tx 2) (2 × 10^10^ CFU) of the probiotic *B. breve* Bif11 MTCC25246 and *L. plantarum* LAB31 combined with IMOS as a synbiotic in both treatments. Verheijden et al. [38] used GOS and lcFOS (Tx 1) and scFOS and lcFOS (Tx 2), both combined with *B. breve* M16V (BB). Sonoyama et al. [40] used RAF (Tx 1), GOS (Tx 2), FOS (Tx 3) and XOS (Tx 4) prebiotic treatments. Finally, Vos et al. [42] used scGOS/lcFOS (Tx 1) and scGOS/lcFOS (Tx 2). Studies which carried out multiple trials are detailed in Table 2.

### 3.3. Effect of Prebiotic and Synbiotic Treatment on Murine Cytokines, Inflammatory Cells and Asthma Outcome Measures

#### 3.3.1. Airway Hyperresponsiveness

**Figure 4:**
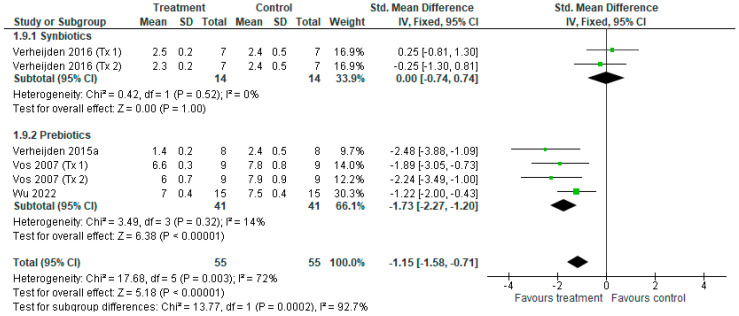
Forest plot of murine studies investigating the effects of synbiotic and prebiotic treatment on airway hyperresponsiveness (AHR), grouped by treatments [15,38,39,42]. Black diamonds indicate the pooled effects of AHR, while green squares and horizontal lines represent the individual study effect sizes and 95% CIs respectively. Standardised mean difference and 95% CIs were calculated utilising an inverse variance fixed effects model, and *I*^2^ values were significant at *p* < 0.05.

For airway hyperresponsiveness (AHR), six treatments from four studies [15,38,39,42] contributed data to meta-analysis (Figure 4). The overall effect of prebiotic and synbiotic treatment revealed a reduction in AHR (SMD = −1.15 [−1.58, −0.71], *p* < 0.001), with considerable heterogeneity among treatments (*I*^2^ = 72%, *p* < 0.05) (Figure 4). Subgroup analysis revealed the synbiotic treatment did not reduce AHR (SMD = 0 [−0.74, 0.74], *p* = 1.0), with no heterogeneity among treatments (*I*^2^ = 0%, *p* = 0.52), whereas prebiotic treatment alone reduced AHR (SMD = −1.73 [−2.27, −1.20], *p* < 0.001), with no heterogeneity (*I*^2^ = 14%, *p* = 0.32). Moreover, a difference between subgroups was found, favouring prebiotic with a moderate to large reduction in AHR over synbiotic treatment for AHR (*p* < 0.001).

#### 3.3.2. Eosinophil Cell Counts

**Figure 5:**
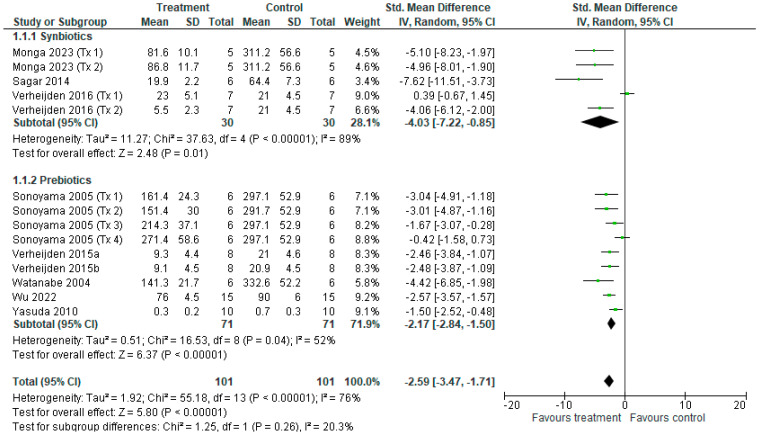
Forest plot of murine studies investigating the effects of synbiotic and prebiotic treatment on BALF eosinophil counts, sub-grouped by treatment [14,15,16,37,38,39,40,41]. The pooled effects are shown as black diamonds, while green squares and horizontal lines represent the individual study effect sizes and 95% CIs respectively. Standard mean difference and 95% CIs were calculated using an inverse variance random effects models, and *I*^2^ values were significant *p* < 0.05.

For BALF eosinophil cell counts, fourteen different treatments from nine studies [14,15,16,37,38,39,40,41,43] contributed to the meta-analysis (Figure 5). The overall effect of synbiotic and prebiotic revealed a reduction in total eosinophil counts (SMD = −2.59 [−3.47, −1.71], *p* < 0.001), with considerable significant heterogeneity among treatments (*I*^2^ = 76%, *p* < 0.001). Synbiotic subgroup analysis revealed eosinophil count reduced following synbiotics (SMD = −4.03 [−7.22, −0.85], *p* < 0.05), with considerable heterogeneity among treatments (*I*^2^ = 89%, *p* < 0.01), and prebiotics (SMD = −2.17 [−2.84, −1.5], *p* < 0.001), with significant heterogeneity among treatments (*I*^2^ = 52%, *p* < 0.05). There was no difference between subgroups (*p* = 0.26).

#### 3.3.3. Neutrophil Cell Counts

**Figure 6:**
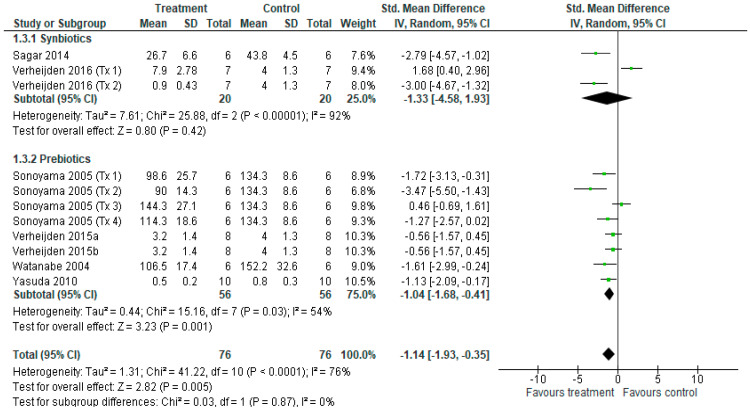
Forest plot of randomised controlled studies investigating the effects of synbiotic and prebiotic treatment on BALF neutrophil counts, sub-grouped by treatment method [14,15,16,38,40,41,43]. The pooled effects are shown as black diamonds and green squares and horizontal lines represent the individual study effect sizes and 95% CIs respectively.. Standard mean difference and 95% CIs were calculated using an inverse variance random effects models, and *I*^2^ values were significant *p* < 0.05.

For BALF neutrophil cell counts, eleven different treatments from seven studies [14,15,16,38,40,41,43] contributed to the meta-analysis (Figure 6). The overall effect of the synbiotic and prebiotic treatments revealed a reduction in total neutrophil counts (SMD = −1.14 [−1.93, −0.35], *p* < 0.05), with considerable heterogeneity among treatments (*I*^2^ = 76%, *p* < 0.001). Subgroup analysis revealed that neutrophil counts were not reduced by synbiotics (*p* = 0.42), with considerable heterogeneity among treatments (*I*^2^ = 92%, *p* < 0.001), but were reduced after prebiotics (*p* < 0.05), with moderate heterogeneity among treatments (*I*^2^ = 54%, *p* < 0.05). Despite a significant effect of prebiotics compared to synbiotics, there was no difference between subgroups (*p* = 0.87).

#### 3.3.4. Alveolar Macrophage Cell Counts

**Figure 7:**
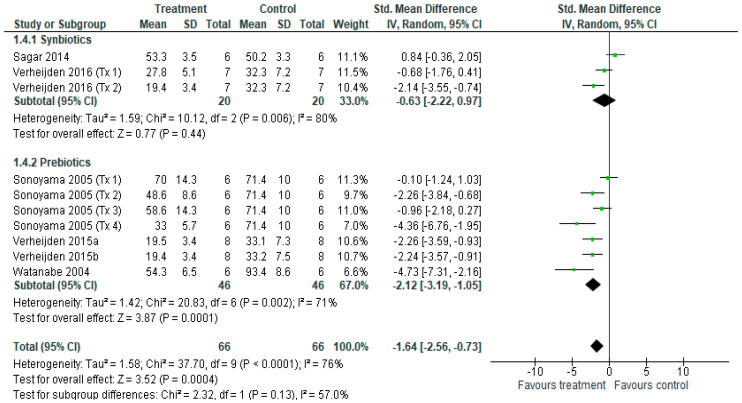
Forest plot of murine studies investigating the effects of synbiotic and prebiotic treatment on BALF alveolar macrophage cell counts, sub-grouped by treatment [14,15,16,38,40,43]. The pooled effects are shown as black diamonds, while green squares and horizontal lines represent the individual study effect sizes and 95% CIs respectively. Standard mean difference and 95% CIs were calculated using an inverse variance random effects models, and *I*^2^ values were significant *p* < 0.05.

For BALF alveolar macrophage cell counts, ten treatments from six studies [14,15,16,38,40,43] contributed to the meta-analysis (Figure 7). The overall effect of synbiotic and prebiotic treatment revealed a reduction in alveolar macrophage cell counts (SMD = −1.64 [−2.56, −0.73], *p* < 0.001), with considerable heterogeneity among studies (*I*^2^ = 76%, *p* < 0.001). Subgroup analysis revealed that alveolar macrophage cell counts were not reduced after synbiotic treatment (*p* = 0.44), with considerable heterogeneity between treatments (*I*^2^ = 80%, *p* < 0.01), but were after prebiotic treatment (*p* < 0.001), with considerable heterogeneity between treatments (*I*^2^ = 76%, *p* < 0.05). There was no significant difference between subgroups (*p* = 0.13).

Antibodies and markers of systemic inflammation.

#### 3.3.5. IL-4 Concentration

**Figure 8:**
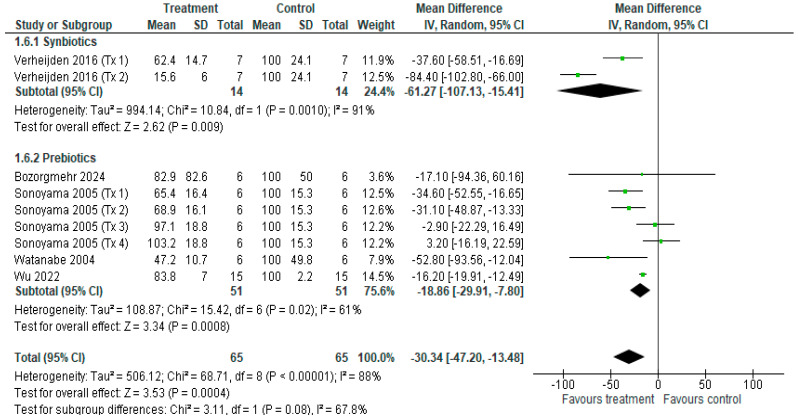
Forest plot of murine studies investigating the effects of synbiotic and prebiotic treatment on IL-4 concentrations, sub-grouped by treatment [14,36,38,39,40]. The pooled effects are shown as black diamonds, while green squares and horizontal lines represent the individual study effect sizes and 95% CIs respectively. Mean difference and 95% CIs were calculated using an inverse variance random effects models, and *I*^2^ values were significant *p* < 0.05.

Nine treatments from five studies [14,36,38,39,40] contributed to the lung IL-4 data for meta-analysis (Figure 8). Overall, compared to the control, there was a reduction in the concentration of IL-4 after synbiotic and prebiotic treatments (MD = −1.51% [−2.40, −0.62], *p* < 0.001), with significant heterogeneity (*I*^2^ = 76%; *p* < 0.05). When evaluated individually, both synbiotic treatment (MD = −2.98% [–5.64, −0.31], *p* < 0.05) and prebiotic treatment (MD = −1.18% [−2.12, −0.24], *p* < 0.05) reduced IL-4 concentrations, with considerable heterogeneity among treatments for synbiotics (*I*^2^ = 77%, *p* < 0.05) and prebiotics (*I*^2^ = 75%, *p* < 0.05 respectively). No significant subgroup differences were found (*p* = 0.08).

#### 3.3.6. IL-5 Concentration

**Figure 9:**
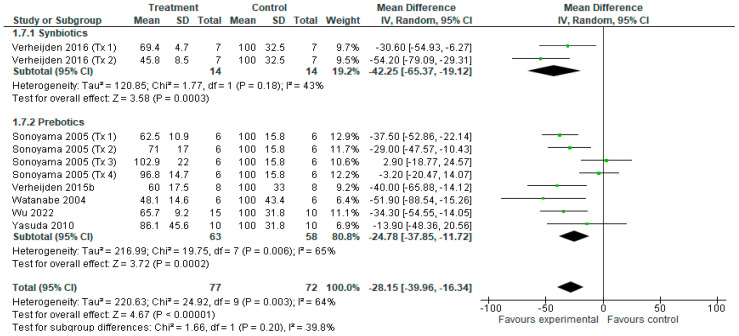
Forest plot of r murine studies investigating the effects of synbiotic and prebiotic treatment on IL-5 concentrations, sub-grouped by treatment [14,38,39,40,41,43]. The pooled effects are shown as black diamonds, while green squares and horizontal lines represent the individual study effect sizes and 95% CIs respectively. Mean difference and 95% CIs were calculated using an inverse variance random effects models, and *I*^2^ values were significant *p* < 0.05.

Ten treatments from six studies [14,38,39,40,41,43] contributed to the lung IL-5 data for meta-analysis (Figure 9). Overall, compared to the control, there was a reduction in IL-5 concentrations after synbiotic and prebiotic treatment (MD = −1.14 [−1.62, −0.44], *p* < 0.001), with small heterogeneity amongst treatments (*I*^2^ = 52%, *p* < 0.05). When evaluated individually, synbiotics (MD = −1.61 [−2.51, −0.70], *p* < 0.001) reduced IL-5 concentrations, with no heterogeneity amongst treatments (*I*^2^ =0%, *p* = 0.33), as did prebiotics (MD = −1.03 [−1.62, −0.44], *p* < 0.001), but with significant heterogeneity across treatments (*I*^2^ = 52%, *p* < 0.05). No subgroup differences were found (*p* = 0.20).

#### 3.3.7. IL-13 Concentration

**Figure 10:**
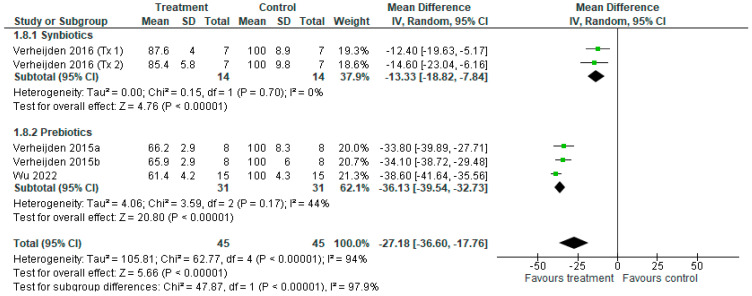
Forest plot of murine studies investigating the effects of synbiotic and prebiotic treatment on IL-13 concentrations, sub-grouped by treatment [15,38,39,40,43]. The pooled effects are shown as black diamonds, while green squares and horizontal lines represent the individual study effect sizes and 95% CIs respectively. Mean difference and 95% CIs were calculated using an inverse variance random effects models, and *I*^2^ values were significant *p* <0.05.

Five treatments from five studies [15,38,39,40,43] contributed to the IL-13 data for the meta-analysis (Figure 10). Overall, compared to the control, there was a reduction in IL-13 mRNA after synbiotic and prebiotic treatment (MD = −4.55% [−7.62, −1.48] *p* < 0.05), with heterogeneity (*I*^2^ = 92% *p* < 0.001). Individually, both synbiotics (MD = −1.69% [−2.96, −0.40] *p* < 0.001) and prebiotics (MD = −7.98% [−9.91, −6.04] *p* < 0.001) reduced IL-13 concentrations, with heterogeneity amongst treatments following the synbiotic (*I*^2^ = 0%, *p* = 0.99) or prebiotic treatment (*I*^2^ = 4%, *p* = 0.31). There were no differences between the subgroups (*p* = 0.2).

#### 3.3.8. Total BALF Cell Count

**Figure 11:**
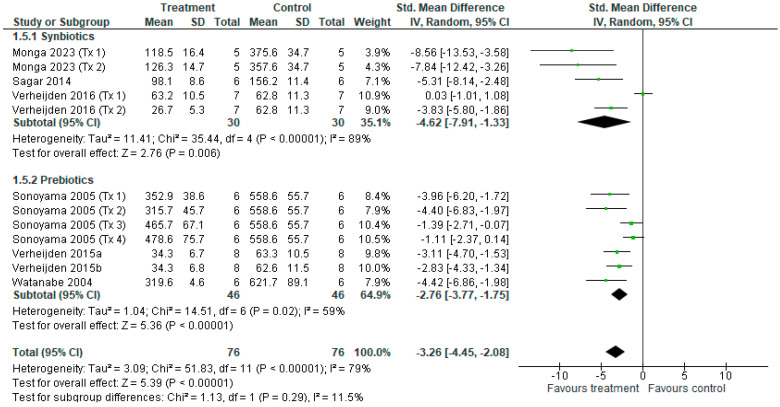
Forest plot of murine studies investigating the effects of synbiotic and prebiotic treatment on total bronchoalveolar lavage fluid (BALF) cell count, sub-grouped by treatment [14,15,16,37,38,40,43]. The pooled effects of BALF cell count are represented as black diamonds, while green squares and horizontal lines represent the individual study effect sizes and 95% CIs respectively. Standardised mean difference and 95% CIs were calculated using inverse variance random effects models, and *I*^2^ values were significant at *p* < 0.05.

Twelve treatments from seven studies [14,15,16,37,38,40,43]. Watanabe et al. [14] contributed to the total BALF cell count data for the meta-analysis (Figure 11). Overall, there was a reduction in total BALF cell count after synbiotic and prebiotic treatment (SMD = −3.26% [−4.45, −2.08] *p* < 0.001) with considerable heterogeneity (*I*^2^ = 79%; *p* < 0.001). When evaluated individually, there was a reduction in BALF cell count following synbiotics (SMD = − 4.62% [−7.91, −1.33] *p* < 0.05) with considerable heterogeneity (*I*^2^ = 89%, *p* < 0.001) and prebiotics (SMD = −2.76% [−3.77, −1.75] *p* < 0.001) with moderate heterogeneity (*I*^2^ = 59%, *p* < 0.05). There were no differences between subgroups (*p* = 0.29).

#### 3.3.9. Lymphocyte Cell Counts

**Figure 12:**
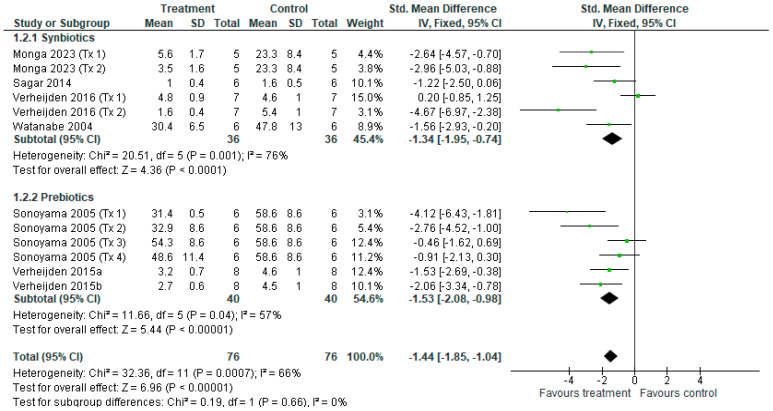
Forest plot of murine studies investigating the effects of synbiotic and prebiotic treatment on BALF lymphocyte counts, sub-grouped by treatment [14,15,16,37,38,40,43]. The pooled effects are represented as black diamonds, while green squares and horizontal lines represent the individual study effect sizes and 95% CIs respectively. Standardised mean difference and 95% CIs were calculated using inverse variance fixed effects models, and *I*^2^ values were significant at *p* < 0.05.

Twelve treatments from seven studies [14,15,16,37,38,40,43] contributed to the total BALF lymphocyte cell count data for the meta-analysis (Figure 12). Overall, there was a reduction in total lymphocyte cell counts after synbiotic and prebiotic treatment (SMD—1.44% [−1.85, −1.04], *p* < 0.001), with significant heterogeneity (*I*^2^ = 66%; *p* < 0.001). When evaluated individually, both synbiotics (SMD—1.34% [−1.95, −0.74], *p* < 0.001) and prebiotics (SMD −1.53% [ −2.08, −0.98], *p* < 0.001) reduced lymphocyte cell counts, with considerate heterogeneity reported following synbiotic treatment (*I*^2^ = 76% *p* < 0.05) and moderate heterogeneity reported following prebiotic treatment (*I*^2^ = 57% *p* < 0.05). There was no difference between subgroups (*p* = 0.66).

### 3.4. Narrative Review—Human Studies

This systematic review provides novel insights into the effects of prebiotics and synbiotics on asthma symptoms and inflammation. It also highlights a significant gap in the literature regarding human in vivo studies. Moreover, the heterogeneity among the study methodologies and subsequent outcomes of available human RCTs in this field highlights the need for additional rigorous studies. A narrative synthesis was performed for the analysis of human studies, as a meta-analysis was not feasible.

#### 3.4.1. Characteristics of Included Studies

Six human RCTs met the eligibility criteria and were included for a narrative review (Table 3). A total of 120 adults and 96 children (≤12 years old) with asthma, aged 13 months–84 years, were included across the studies. The six studies were carried out across varied geographical regions, including Iran, Australia, and England, between 2010 and 2025. Sample sizes ranged from 10 to 100 per RCT. Across the six RCTs, different prebiotics and synbiotics were administered with treatment periods ranging from 1 acute dose up to 6 months. A summary of all human RCTs is shown in Table 3.

#### 3.4.2. Intervention Characteristics

The type of prebiotic and synbiotic varied considerably between RCTs. Two human studies evaluated the effect of prebiotics alone. Williams et al. (2016) provided a daily dose of 5.5 g.d^−1^ Bimuno-galactooligosaccharides for three weeks [24] and Berthon et al. [45] provided a daily dose of an inulin (1 × 6 g/1 × 12 g/2 × 6 g, daily), FOS, and GOS mixture for 14 days. Across all trials, carbohydrate-based prebiotics were used. Regarding synbiotic use, studies provided various combinations, including prebiotics such as short-chain galactooligosaccharides, long-chain fructooligosaccharides, fructooligosaccharides, or inulin paired with probiotic strains such as *Bifidobacterium Breve M-16V*, *Bifidobacterium infantis*, *Lactobacillus acidophilus*, *Lactobacillus* spp., *Bifidobacterium* spp., *Streptococcus thermophiles*, and *Lactobacillus rhamnosus* [23,25,30,44].

The duration of prebiotic treatment ranged from one single dose, up to 6 months of daily treatment. The prebiotic treatments in two studies were administered in a powdered form [24,45], whilst three synbiotic studies varied between powder and capsule form [23,25,30]. The final synbiotic study treated the synbiotic within a yoghurt in a single meal [44].

Concomitant treatments included appropriate steroid medications [24,25,30]. Attempts were made to control for concomitant medication, but this varied across studies; in Williams et al.’s [24] study, participants were instructed to stop taking their medication (inhaled corticosteroids: 4 d; inhaled long-acting β2 agonists: 2 d; inhaled short-acting β2 agonists: the day of the test) prior to each visit, and in other studies, participants were instructed to withhold short-acting β2-agonist medications for 12 h and long-acting β2-agonist medications for 24 h before study days [25,44]. Van de Pol and colleagues [23] instructed participants to stop using short-acting β2- agonists for at least 12 h before each visit and long-acting β2-agonists, oral antihistamines, and inhaled corticosteroids for 4 weeks prior to and during the study [23]. No control over asthma medication was made in the final two studies [30,45].

Treatment compliance was difficult to assess, as participants were required to consume the treatment at home, although techniques such as pill/sachet countback, daily food diaries and recall, and questionnaires were used to monitor adherence. Minimal data on habitual diet was collected.

#### 3.4.3. Reported Outcome Measures

One study primarily investigated self-reported asthma-associated measures, including the number of hospitalisation visits, frequency of asthma exacerbations and satisfaction, and side effects of the interventions [25]. Two studies measured asthma control via the ACQ-6 questionnaire [30] and the ACQ7 questionnaire [45]. Four studies in this narrative review assessed various objective, measures including airway inflammation, inflammatory responses, lung function [23,24,45], and microbiota composition [30]. Mcloughlin et al. [30], Berthon et al. [45], and Halnes et al. [44] additionally assessed SCFA profiles, and GPR41 and GPR43 expression were assessed by Halnes et al. [44] and Mcloughlin et al. [30].

#### 3.4.4. Systemic Inflammation Outcome Measures

Systemic inflammatory biomarkers were assessed in three human RCTs [23,24,45] whilst the remaining studies did not take measurements of systemic inflammation following prebiotic and synbiotic intervention [25,30,44].

In response to a bronchoprovocation challenge, Williams et al. [24] demonstrated that a 21-day prebiotic intervention (GOS) abolished the increase in TNF-α (*p* = 0.002) compared to day 0. Moreover, baseline CCL17 (339 ± 140 pg/mL vs. 323 ± 144 pg/mL, *p* = 0.005), CRP (2.46 ± 1.14 mg/L vs. 1.44 ± 0.41 mg/L, *p* = 0.015) and TNF-α (2.68 ± 0.98 pg/mL vs. 2.18 ± 0.590 pg/mL, *p* = 0.04) were reduced in the physically active asthma participants after 21 days of B-GOS intervention [24]. Van de Pol and colleagues [23] additionally reported statistically significant reductions in serum IL-5 following the synbiotic intervention compared to the placebo group after exposure to a HDM allergen challenge (*p* = 0.034). Finally, Berthon et al. (2025) reported within-arm reductions in blood eosinophils, neutrophils and lymphocytes following the low-dose prebiotics intervention [45].

#### 3.4.5. Airway Inflammation Outcome Measures

Five out of the six included RCTs reported outcome measures related directly to airway inflammation. Van de Pol et al. [23] assessed inflammatory responses to a HDM bronchial provocation challenge. Following the synbiotic intervention, the increase in both in vivo and ex vivo cytokine IL-5 production was reduced (*p* = 0.034), as was the ex vivo type 2 cytokine IL-4 and IL-13 production from stimulated peripheral blood mononuclear cells (PBMC) (*p* = 0.046). No changes in the fraction of exhaled nitric oxide (FeNO) were reported following the synbiotic study [23]. Mcloughlin et al. [30] additionally reported a significant reduction in sputum %eosinophil (*p* = 0.006) following the prebiotic (inulin) intervention. Although not significant, Berthon and colleagues (2025) also reported a trend in the reduction in sputum IL4, IL-5 and IL13 in eosinophilic asthma following a low prebiotic dose [45].

Halnes et al. [44] reported reductions in sputum neutrophils (*p* = 0.033), macrophages *(p* = 0.030), lymphocytes (*p* = 0.002) and sputum IL-8 (*p =* 0.005) 4 h post a single soluble fibre meal. This corresponded with upregulated GPR41 (*p* = 0.027) and GPR43 sputum expression (*p* = 0.007) in the soluble fibre group [44]. The remaining study reported no significant changes of IgE and FeNO following prebiotic treatment [24].

#### 3.4.6. Lung Function Outcome Measures

Five RCTs reported pulmonary function data. In Williams et al. [24], the severity of hyperpnoea-induced bronchoconstriction was reduced following 21 days of B-GOS. Specifically, the fall in FEV_1_ after bronchoprovocation was attenuated by 40% from day 0 (−940 (SD 460) mL) to day 21 (−570 (SD 310) mL) (mean difference = 370 (SD 290) mL; 95% CI 166, 575 mL, *p* = 0.004). Halnes et al. [44] reported increases in lung function 4 h following a single synbiotic fibre meal, including an improvement in FEV_1_ by 0.1 L (median 2.7 L to 2.8 L) and an increase in FEV_1_/FVC by 3.8%. However, these changes did not reach statistical significance (*p* = 0.002). Morning and evening peak expiratory flow improved over 4 weeks of a synbiotic intervention (morning *p* = 0.003, evening *p* = 0.011) compared to placebo, but there was no change in airway hyperresponsiveness to a house dust mite (HDM) allergen challenge [23]. The remaining two RCTs reported no changes in lung function (FEV_1_) following 7 days of synbiotic or prebiotic treatment compared with placebo [30] and 14 days of prebiotics compared with placebo [45].

#### 3.4.7. Outcomes Regarding Asthma Control and Symptoms

Two RCTs reported measures of asthma control and one RCT reported the frequency of asthma exacerbations and hospital visits. Of those, Mcloughlin et al. [30] reported an improvement in asthma control following the prebiotic-only intervention, indicated by a reduction in ACQ-6 questionnaire scores (*p* = 0.006) in all participants. The reduction in ACQ-6 scores reached clinical significance in 63% of participants with partial–poorly controlled asthma [30]. Berthon et al. (2025) also reported a significant with-in arm reductions in ACQ-7 scores following the low prebiotic arm [45]. Hassanzad et al. [25] reported reduced outpatient visits following the synbiotic treatment compared with placebo (19 and 55 visits, respectively), but no significant changes were observed in frequency of asthma attacks and hospital visits due to exacerbation.

In relation to adverse events, one study reported an increase in diarrhoea following a synbiotic-only treatment (*n* = 1) and increase in both diarrhoea and discomfort from indigestion following a prebiotic-only treatment (*n* = 3) [23]. Hassanzad et al. [25] reported that 19 participants withdrew: 5 from the synbiotic group (including 1 due to vomiting), and the remaining 14 withdrew from the placebo group due to various effects including vomiting, headache, stomach-ache and diarrhoea, exacerbated cough, and severe constipation. Additionally, Berthon et al. (2025) reported one event of GI symptoms in the 2 × 6 g and 1 × 12 g daily dose [45].

## 4. Discussion

To our knowledge, no systematic review or meta-analysis has summarised the effects of prebiotic and synbiotic treatment on asthma management. Despite limited human studies, this narrative review highlights benefits of prebiotics and synbiotics on asthma associated outcomes, including improvements in lung function, reduced airway hyperresponsiveness, enhanced asthma control, and reductions in circulating inflammatory cells. Given the considerable intervention heterogeneity across murine and human studies, including variations in dose, duration, treatment type, and control of concomitant medication, alongside the limited number of available trials, the direct clinical translation of these findings remains limited. Nonetheless, the meta-analysis of murine studies reveals positive mechanistic evidence supporting the notion that prebiotics and synbiotics may be a suitable adjunct treatment for asthma. Prebiotic treatment reduced all measured asthma-related parameters in murine models, whereas synbiotics selectively reduced total BALF cell count, BALF lymphocyte and eosinophil cell counts, and lung IL-4, IL-5, and IL-13 concentrations.

The evidence for health benefits of prebiotics is growing and highlights anti-inflammatory and immunomodulatory effects in atopic disease, with some evidence suggesting enhanced efficacy when combined with probiotics as synergistic synbiotics [46]. However, the present meta-analysis of murine asthma models found greater improvements in asthma-related outcomes with prebiotics alone compared to synbiotics. This aligns with prior findings by McLoughlin et al. [30], where only 43% of studies reported superior anti-inflammatory effects from synbiotics over prebiotics alone. The observed discrepancy may result from the suboptimal pairing of prebiotics and probiotics within current synbiotic formulations. It must be acknowledged that the significant heterogeneity in dosage and substrate strains may confound direct comparisons and future research utilising standardised dosages and rational pairing of synergistic synbiotics is warranted.

Across the murine trials, prebiotic subgroup meta-analysis indicated that GOS was the most effective prebiotic for reducing airway hyperresponsiveness and neutrophil counts [14,38,40], supporting a previous meta-analysis that demonstrates a superior reduction in systemic inflammation following GOS supplementation [31]. GOS has been shown to promote greater growth of beneficial bacteria such as *Lactobacillus* and *Bifidobacterium* compared to ROS [46,47]. The subsequent eubiosis of the gut microbiome facilitates microbial fermentation of oligosaccharides into anti-inflammatory short-chain fatty acids (SCFAs), specifically butyrate, acetate, and propionate [48]. The association between prebiotics and attenuated type 2 inflammation is largely mediated by SCFAs, which enter systemic circulation and reach peripheral tissues. Upon reaching the airways, these metabolites reduce inflammatory mediators, including neutrophils and leukocytes, via histone deacetylase (HDAC) inhibition, consequently promoting regulatory T cell (Treg) expansion and activity [49,50]. Furthermore, systemically circulated SCFAs serve as ligands for GPR41 and GPR43 receptors on airway immune cells, which subsequently suppress the production of pro-inflammatory cytokines including TNF -α, IL-6, and IL-8 [44,46].

Prebiotics and synbiotics, including the genera of *Bifidobacteria* and *Lactobacillus*, stimulate the growth of beneficial bacteria in the colon [30]. These commensals are widely recognised for their antagonistic action against pathogenic microorganisms and implications in immune disease modulation [37]. *Bifidobacteria* and *Lactobacillus* indirectly regulate inflammation through supporting the repair of hyperpermeable epithelial barriers and attenuating the damaging actions of pathogenic microorganisms. As transports of pathogenic microorganisms across the epithelial barrier becomes limited, their capacity to act as an additional trigger in the inflammatory cascade reduces [51]. Beneficial bacteria can increase the synthesis of anti-microbial peptides involved in key local pathways and directly influence immune cells including dendritic cells and T cells [52] and regulatory cytokines, including IL-10 and TGF-β [53]. Furthermore, commensal bacteria and their by-products can modulate the inflammatory response by acting as ligands for innate immune system receptors and directly modulate pro-inflammatory pathways, including the NF-kB and mitogen-activated protein kinase (M-APK) pathways [52].

Collectively, the immunomodulatory actions are critical for the reduction in chronic airway inflammation and the hyperresponsiveness characteristic in asthma.

Raffinose, an oligosaccharide found in lentils, legumes, and soybeans, has recently gained interest for its anti-inflammatory benefits. Raffinose has been shown to increase beneficial bacteria and SCFA production while reducing proteobacteria, a group of pathogenic bacteria implicated in asthma [53]. The present subgroup prebiotic meta-analysis revealed that raffinose was the most effective treatment for reducing BALF cell counts, IL-4, IL-5, BALF eosinophil and alveolar macrophage counts, with all data derived from Watanabe et al.’s [14] study. The benefits of raffinose have been attributed to its α-D-galactosidic linkages [54], which regulate immune functions via natural killer T cell activation. Despite encouraging findings, Sonoyama et al.’s [40] study, a direct comparison of RAF, GOS, FOS, and XOS, found raffinose to be least effective amongst the trials in reducing alveolar macrophages. Raffinose remains a relatively new prebiotic, and the inconsistency in findings highlights the requirement of further mechanistic and interventional studies to fully understand its implications for health.

GOS, derived from the action of β-galactosidase on lactose [54], and FOS, which is found in artichoke, chicory, and asparagus [55], are oligosaccharides recognised for their ability to promote the growth of beneficial bacteria, including *Bifidobacteria* [56]. In the present murine meta-analysis, the combination of prebiotics GOS and FOS as a synbiotic, with various probiotics, demonstrated varying effects on murine asthma parameters. The GOS/FOS and *B. breve* M16V combination [38] (Tx 2) most effectively reduced IL-4, AHR, and lymphocyte counts. For other outcomes, different synbiotic formulations were more effective: scFOS/lcFOS/AOS with *B. breve* M-16V [16] most effectively reduced eosinophil counts, while FOS with *B. breve* M-16V (Tx2) most effectively reduced neutrophil counts, alveolar macrophages, IL-5, and IL-13. Total BALF cell count was most effectively reduced by IMOS *B. breve* Bif11 and *L. plantarum* synbiotic [37] (Tx 1 and Tx 2). Variations in the composition and purity of commercial synbiotics across studies [57] may contribute to the observed inconsistency in synbiotic effects. Future studies should consider combining standardised prebiotics with known compositions and purity with synergistic probiotics to formulate more optimal synbiotics.

The present meta-analysis indicates that interventions combining pectin-derived acidic oligosaccharides with neutral oligosaccharides [16] were more effective in reducing BALF eosinophil counts than interventions using neutral oligosaccharides alone. Further, Vos et al. [42] reported greater reductions in murine airway hyperresponsiveness when GOS and FOS were combined with AOS (Tx 1) compared to GOS and FOS (Tx 2) alone, demonstrated in the present meta-analysis. Supporting these findings, an in vitro study by Eiwegger and colleagues [58] showed that AOS increased CD4+ and CD8+ T cells and cytokine production in cord blood mononuclear cells compared to neutral oligosaccharides. Pectin-derived acidic oligosaccharides have gained attention for their ability to inhibit the binding of pathogenic bacteria to epithelial cells [59] and improve the Th1-type response [42], offering a mechanistic explanation for their anti-inflammatory effects. However, research on AOS treatment to modulate inflammation is limited, and additional studies are warranted to fully establish the effects and translational implications of adding AOS to neutral prebiotics.

Regarding the narrative review on human intervention studies, one trial reported improvements in exercise-induced bronchoconstriction following a 3-week GOS prebiotic intervention. This was shown by reductions in the fall in FEV_1_ and PEF (peak expiratory flow) fall, following a bronchoprovocation challenge [24]. Additionally, despite Van de Pol et al. (2011) [23] reporting no change in airway hyperresponsiveness, participants demonstrated improvements in daily lung function, assessed by resting morning and evening PEF [23], following 4 weeks of supplementation. Mcloughlin et al. [30] and Berthon et al. [45] reported no significant improvements in lung function following a relatively shorter 7-day and 14-day prebiotic and synbiotic intervention, respectively. Differences in the findings may be explained by the metabolic activity and interaction of the prebiotic with commensal bacteria to produce beneficial SCFA metabolites. Although SCFAs are widely recognised for their anti-inflammatory properties in various diseases, including asthma [30], these metabolites have not been detectable in the lungs following soluble fibre treatment in murine or human studies [60]. It is therefore likely that some anti-inflammatory effects of prebiotics and synbiotics occur systemically [44], and longer durations of treatment may be required to achieve greater anti-inflammatory effects in the lungs [30].

Across human trials, there is high heterogeneity and insufficient data on subjective reporting of asthma control and disease management. One human study reported a significant reduction in the need for outpatient visits between a synbiotic group (19 visits) and a control group (55 visits) and reported fewer side effects and significantly greater satisfaction with treatment in the synbiotic group compared to the control group [25]. However, no significant difference in the frequency of asthma exacerbations and hospitalisations due to asthma exacerbations were observed [25]. Another study reported a clinically meaningful 63% improvement in ACQ-6 scores following prebiotic treatment, with no effect following synbiotic treatment [30]. However, in the same study, participants reported more indigestion and discomfort following prebiotic treatment, including a higher incidence of diarrhoea following the synbiotic treatment, compared to baseline. Berthon and colleagues (2025) reported no improvements in ACQ-7 scores between the prebiotic and placebo, although within-arm significance was reported following a low dose of the prebiotic. While this data is promising, additional studies are warranted to understand the benefits of prebiotics and synbiotics on perceived asthma and should consider the influence of participant’s asthma severity on treatment efficacy [30].

This systematic review was limited by the significant heterogeneity across the included studies, particularly regarding the specific types, doses, and intervention durations of prebiotic and synbiotic formulations. Furthermore, the lack of standardised pairing between specific probiotic strains and prebiotic substrates complicates direct relational comparisons. These variations limit interpretations of an ‘optimal’ synbiotic formulation for asthma. Additionally, since the present analysis combines both adult and child interventions, varying effects of age on microbiome modulation should be considered when interpreting findings. Regarding murine studies, the significant heterogeneity of the animal models of AAI and cytokine sources may impede the contribution of our findings and contribute to the difficulty in providing clear translation to human intervention trials. Whilst the authors of murine studies were contacted for raw data, most did not respond, limiting the data included in the murine meta-analysis. The lack of information on human habitual diet caused ambiguity in the interpretation of the results. Finally, all human studies in this review evaluated prebiotic and synbiotic treatment for ≤6 months, limiting our understanding of long-term human interventions. Future research should consider direct comparisons of prebiotic and probiotic combinations to determine the appropriate doses and investigate longer treatment durations to establish optimal supplement duration.

Despite these limitations, to our knowledge, this is the first review to systematically examine the available evidence of the effect of prebiotics and synbiotics on asthma outcomes and extrapolate generalisations which bridge the gap between murine models and human participants. Furthermore, we performed subgroup analysis for prebiotics and synbiotics, coupled with a combined analysis, to further our understanding of the overall benefit of prebiotics and synbiotics on asthma outcomes. Finally, we followed a pre-defined review protocol, which was registered before we began our search.

## 5. Conclusions

Collectively, while animal models and a limited number of human studies provide preliminary evidence that prebiotics and synbiotics may contribute to asthma modulation, current findings warrant further translational investigation. Although current murine data offer a plausible mechanistic basis for gut-lung modulation in asthma, the available human evidence remains constrained by significant heterogeneity and short trial durations. Consequently, to evaluate clinical translational potential, additional robust human trials are warranted to determine whether the immunomodulatory effects observed in pre-clinical models can be replicated as meaningful clinical outcomes in patients diagnosed with asthma.

## Figures and Tables

**Table 2:** Study characteristics and intervention outcomes of murine studies included in the meta-analysis.

Study	Murine Strain	Murine (*n*)	Intervention	Control	Dose and Description of Treatment	Duration of Intervention/Sensitisation	Outcome of Intervention	Specimen Type
Prebiotic interventions								
Bozorgmehr et al., 2024 [36]	C57BL/6J mice	6	Prebiotic	Phosphate-buffered saline replacing all HMOs	2′-fucosyllactose (2′-FL) and 6′-sialyllactose (6′-SL), 500 mg/kg daily from day 7 to 21.	HDM 15 days (1 μg of HDM protein and 40 μL PBS on day 49 to 53)	Reduction in IgE, eosinophils, neutrophils (cells/mL BALF 10^6^). Reduction in IL-4 and IL-6 in lung tissue.	BALF absolute cell count Lung tissue (pg/g tissue)
Sonoyama et al., 2005 [40]	Brown, Norway rats	6	Prebiotic	AIN93G control diet. Ad libitum feeding.	Tx 1: FOS, Tx 2: GOS, Tx 3: RAF, Tx 4: XOS, 7 days (50 g/kg of diet) or injected for 13 days (0.5% daily [wt:v]).	OVA 7–13 days (1 mg and 0.2 mL Bordetella Pertussis vaccine)	Reduced airway eosinophilia. Reduced inflammatory cells in BALF. RAF—Reduced IL-5, associated with reduced IL-4 and IL-5 in Lung tissue.	Lung tissue (mRNA relative fold change) BALF (absolute cell count)
Verheijden et al., 2015a [43]	BALB/C	8	Prebiotic	AIN93G control diet. Control separately treated with budesonide (oropharyngeal, 500 µg/kg) on days 7,9 and 11 prior to daily challenge and day 13	GOS (1 *w*/*w*%) Vivinal GOS syrup (1% *v*/*w*): 21% Lactose, 19% Glucose, 59% GOS and 1% Galactose on dry matter (75%).	HDM 14 days (intranasal 1 µg on day 0)	1% GOS prevented development of AHR. Reduced CCL5 and IL-33. Reduced airway eosinophilia.	Lung tissue (mRNA relative fold change) BALF (absolute cell count)
Verheijden et al., 2015b [15]	BALB/C	8	Prebiotic	Ad libitum feeding of AIN93G control diet	Vivinal GOS (1% *v*/*w*) syrup: 21% Lactose, 19% Glucose, 59% GOS and 1% Galactose on dry matter (75%).	HDM 14 days (intranasal 1 µg on day 0)	Reduced IL-33 mRNA associated with average BALF cell count and intestinal permeability. Reduced IL-33 and ST2 mRNA.	Lung tissue (pg/mg protein) BALF (absolute cell count)
Vos et al., 2007 [42]	BALB/C, CBBYJLO, SPF	9	Prebiotic	All treatment of oligosaccharides replaced carbohydrate content. Semi purified AIN-93G based diets	Tx 1: scGOS/lcFOS.(9:1 1% *w*/*w*). Tx 2: 83% scGOS/lcFOS and 17% pAOS (1% *w*/*w*). scGOS/lcFOS and scGOS/lcFOS/pAOS: 1% (*w*/*w*% net oligosaccharides).	OVA 55 days. 2 10 µg intraperitoneal injections	Airway hyperresponsiveness to Methacholine was significantly reduced in both scGOS/lcFOS and scGOS/lcFOS/paAOS diet groups. Reduced inflammatory cells in BALF. Tx 2: reduced AHR and BALF cell count to a greater extent.	BALF (absolute cell count)
Watanabe et al., 2004 [14]	Brown, Norway rats	6	Prebiotic	Ad libitum feeding of AIN93G control diet	RAF (50 g/kg of diet). 7 days RAF injected daily alongside RAF diet.	OVA 20 days (mg and 0.2 mL Bordetella Pertussis vaccine)	Reduced IL-4 and IL-5 (mRNA) Reduced total BALF cell count and eosinophil %. Reduced airway eosinophilia with intraperitoneal injection of RAF.	Lung tissue (mRNA relative fold change) BALF (absolute cell count)
Yasuda et al., 2010 [41]	C3H/Hen	10	Prebiotic	CNTRL diet (/100 g/diet), 7.7 g moisture. 5.3 g crude fat, 23.6 crude protein, 6.1 g crude carbohydrates, 2.9 g crude fibres, 54.4 g soluble nitrogen free compound. Food and water ad libitum.	FOS fed ad libitum (2.5%) 7 days prior to allergen administration. FOS replaced 2.4% of control diet.	HDM 50 days (*Dermatophagoides farinae*—1 µg)	Reduced IL-5 (mRNA and protein) Reduced IgG and IgE. Reduced eosinophil cell count with FOS following HDM challenge.	Lung tissue (mRNA relative fold change) BALF (absolute cell count)
Synbiotic interventions								
Monga et al., 2023 [37]	BALB/C	5	Synbiotic	Ad libitum pellet diet	Tx 1: Isomaltooligosaccharides (IMOS), 1 g/kg body weight 1 × 10^10^ CFU *B. breve* Bif11 and *Lactiplantibacillus plantarum* LAB31. Tx 2: IMOS and 2 × 10^10^ CFU of each strain. Daily 39 days.	OVA 14 days (10 μg of OVA and 2 mg of Alum on day 0 and 14)	Synbiotics Reduce BALF eosinophils, total cell count and lymphocytes.	BALF (absolute cell count)
Sagar et al., 2014 [16]	BALB/C	6	Synbiotic	CNTRL diet not specified. ‘Standard conditions with free access to food and water’	scFOS/lcFOS/AOS Oral gavage, 0.2 mL of mixture in PBS, day 22–55 3× week. *B breve M-16V* (10^9^ CFU) with maltodextrin as the carrier.	OVA 33 days (10 µg on day 0 and day 12)	Reduced eosinophils Reduced inflammatory cells in BALF. Reduced IL-1β, IL-6, IL-12 and TNF-α in lung tissue mRNA.	BALF (absolute cell count) Lung tissue (mRNA relative fold change)
Verheijden et al., 2016 [38]	BALB/C	7	Synbiotic	Ad libitum feeding of AIN93G control diet	Tx 1 FOS (ScFOS and lcFOS, 1:1) Tx 2 GOS and LcFOS (1% *w*/*w*), 95% oligofructose content. *B. breve* M16V (BB) with maltodextrin as the carrier.	HDM 14 days (intranasal 1 µg on day 0)	Combination of GOS/lcFOS was less favourable than scFOS/ lcFOS and *B. breve* diet. Reduced IL-6, IL-4, IL-10 and IFNγ. No significant changes in AHR response.	BALF (absolute cell count) Lung tissue protein (pg/mL)
Wu et al., 2022 [39]	BALB/C	15	Synbiotic	Phosphate-buffered saline replacing synbiotic.	FOS and GOS 10 mg/kg body weight. Daily from day 15 to day 25 *Lactobacillus acidophilus* LA-5, L. rhamnosus GG, and *B animalis subspecies lactis* BB-12. 25b CFU. Daily from day 15–day 25.	14 days (100 μg 7 1 mg aluminium hydroxide gel, days 1, 7 and 14)	Reduced airway hyperresponsiveness. No significant findings of eosinophilic infiltration. No significant findings of cytokine concentrations.	BALF (absolute cell count)

Abbreviations: HMO’s, human milk oligosaccharides; HDM, house dust mite, BALF cell count, bronchoalveolar lavage fluid; OVA, ovalbumin; PBS, phosphate-buffered saline; CFU, colony forming unit; FOS, fructooligosaccharides; GOS, galactooligosaccharides; RAF, raffinose; XOS xylooligosaccharides; AHR, airway hyperresponsiveness; pAOS, potato-derived acidic oligosaccharides; Tx, Treatment.

**Table 3:** Summary of human studies examining the effects of prebiotics and synbiotics on asthma outcomes.

Study	Study Design	Population	Intervention	Dose and Description of Treatment	Intervention Duration	Outcome Measures
Prebiotic interventions						
Berthon et al., 2025 [45]	Double blind, randomised, placebo controlled, 4 arm crossover trial	35 adults with asthma	Prebiotic	Inulin, FOS, GOS mixture 2 × 6 g daily 1 × 6 g daily 1 × 12 g daily	14 days	With-in arm asthma control increase (AQLQ-7) and sputum count reduction following 1 × 6 g SCFAs increased following 1 × 12 g
Williams et al., 2016 [24]	Double blind RCT, two-way crossover	10 adults with asthma (HIB)	Prebiotic	Bimuno GOS (5.5 g/day active GOS derived from lactose using a beta-galactosidase enzyme) Oral administration (powder form)	3 weeks	Attenuation in FEV1 following EVH challenge Reduction in TNF-α, CCL17, CRP Improvement in PEF
Synbiotic interventions						
Halnes et al., 2017 [44]	RCT, parallel design	29 adults with asthma	Prebiotic and Synbiotic	Probiotic yoghurt (175 g) *Lactobacillus acidophilus* LA5, *Bifidobacterium lactis* Bb12, *Lactobacillus rhamnosus strain* GG, all ≥108 CFU soluble fibre inulin (3.5 g)	Single meal	Improvement in FEV1 and FEV1/FVC ratio Reduction in sputum IL-8 and total cell count Reduction in neutrophils macrophages and lymphocytes Upregulation of sputum cell GPR41 and GPR43
Hassanzad et al., 2019 [25]	Double blind, placebo controlled parallel design	96 children with asthma (phenotype not specified)	Synbiotic	1 sachet/day Kilidact^®^: fructo-oligosaccharide (powder form) with *Lactobacillus casei* *Lactobacillus acidophilus* *Lactobacillus rhamnosus* *Lactobacillus bulgaris* *Bifidobacterium infantis* *Bifidobacterium breve*	6 months	Fewer outpatient visits Higher satisfaction of treatment compared with control Fewer side effects
Mcloughlin et al., 2019 [30]	Double blind RCT, three-way crossover (soluble fibre and probiotic Soluble fibre Placebo)	17 adults with asthma	Synbiotic	Exp 1. Prebiotic: 12 g/day inulin Oral administration (powder form) Exp 2. Synbiotic: 12 g/day Inulin and *Streptococcus thermophiles Lactobacillus rhamnosus GG* *Bifidobacterium animalis (8.5* × *10^9^ CFU)* Exp 3. Placebo: maltodextrin powder	7 days	Improvement in ACQ-6 scores Reduction in % sputum eosinophils Reduced sputum HDAC9 expression Changes in specific bacterial operational taxonomic units in the gut microbiome
van de Pol et al., 2011 [23]	Double blind RCT, parallel design	30 adults with asthma and HDM allergy	Synbiotic	8 g/day scGOS/lcFOS. Oral administration combined with Immunofortis (powder form) and *B. breve* M-16V 10 × 10^10^	4 weeks	Improvement in morning PEF Inhibition of IL-5 following HDM challenge compared to placebo Reduction in IL-4 and IL-13 following HDM compared with placebo

Key: RCT, randomised cross-over trial; HIB, hyperpnoea-induced bronchoconstriction GOS, galacatooligosaccharide; FOS, fructooligosaccharide; EVH, eucapnic voluntary hyperpnoea; PEF, peak expiratory flow; FEV1, forced expiratory volume in 1 s; FVC, Forced Vital Capacity; HDM, house dust mite.

## Data Availability

No new data were created or analysed in this study. Data sharing is not applicable to this article.
